# Correction: Cues to improve antibiotic-allergy registration: A mixed-method study

**DOI:** 10.1371/journal.pone.0293149

**Published:** 2023-10-12

**Authors:** The PLOS ONE Staff

There are errors in the Funding section. The correct Funding statement is: This study was part of a project on improving antibiotic allergy registrations, which was funded by the Dutch Ministry of Health, Welfare and Sport (Reference number 327952).

The publisher apologizes for the error.
