# Supplementary material for: Cues to improve antibiotic-allergy registration: A mixed-method study
Source: PLoS One. 2022 Apr 7;17(4):e0266473. doi: 10.1371/journal.pone.0266473 (PMC8989191; doi:10.1371/journal.pone.0266473)
Supplement: S1 Table — (DOCX) [file pone.0266473.s001.docx]

# S1 Table. Modified checklist of Salden^*^

|  | | Immediate type probable | Immediate type possible | Delayed type probable | Delayed type possible | Immediate/ delayed type Possible | Non-allergic reaction |
| --- | --- | --- | --- | --- | --- | --- | --- |
| Time to symptoms | | < 6 hours | < 6 hours or unclear | > 6 hours | > 6 hours or unclear | > 6 hours or unclear | > 14 days OR any time |
|  | | *AND* | *AND* |  |  |  | *AND* |
| Symptoms | | | | | | | |
|  | Urticaria | Yes and/or | Yes and/or | No | No | Yes and/or | No |
|  | Angio-oedema | Yes and/or | Yes and/or | No | No | No | No |
|  | Rash or exanthema | Yes and/or | Yes and/or | Yes | Yes | Yes and/or | No |
|  | | *AND TWO OF 1-5* | *AND ONE OF 1-3 OR 5* |  |  |  |  |
|  | 1 Dyspnoea | Yes | Yes | No | No | No | No |
|  | 2 Collapse | Yes | Yes | No | No | No | No |
|  | 3 Nausea, vomiting or diarrhoea | Yes | Yes |  |  |  |  |
|  | |  |  | *AND ONE OF 4-5* |  |  | *AND/ OR* |
|  | 4 Repeated reaction when re-exposition to same antibiotic | Yes | No | Yes | No | No | No |
|  | 5 Confirmed by dermatologist/ allergist | Yes | Yes | Yes | No | No | No |

*Checklist is from Salden OA, Rockmann H, Verheij TJ, Broekhuizen BD. Diagnosis of allergy against beta-lactams in primary care: prevalence and diagnostic criteria. Family practice. 2015;32(3):257-62.
