# Supplementary material for: Cues to improve antibiotic-allergy registration: A mixed-method study
Source: PLoS One. 2022 Apr 7;17(4):e0266473. doi: 10.1371/journal.pone.0266473 (PMC8989191; doi:10.1371/journal.pone.0266473)
Supplement: S2 Table — (DOCX) [file pone.0266473.s002.docx]

# S2 Table. COnsolidated criteria for REporting Qualitative research^*^ checklist

| **Topic** | **Item No.** | **Guide Questions/Description** | **Answers** |
| --- | --- | --- | --- |
| **Domain 1: Research team and reﬂexivity** | | | |
| *Personal characteristics* | | | |
| Interviewer/facilitator | 1 | Which author/s conducted the interview or focus group? | Five interviewers (KB, ML, YA, BH and MS) conducted semi-structured interviews |
| Credentials | 2 | What were the researcher’s credentials? E.g. PhD, MD | Karolina K. Braun, MD^1^  Merel M.C. Lambregts, MD^2^  Youssra Atmani, MSc^1^  Bart J.C. Hendriks, MPharm^3^  Martijn Sijbom, MD^4^ |
| Occupation | 3 | What was their occupation at the time of the study? | 1. Medical student 2. Medical specialist 3. Pharmacist 4. Primary care physician |
| Gender | 4 | Was the researcher male or female? | Two were male (M.S. and B.H.). |
| Experience and training | 5 | What experience or training did the researcher have? | Interviewers had training in conducting semi-structured interviews. |
| *Relationship with participants* | | | |
| Relationship established | 6 | Was a relationship established prior to study commencement? | Prior to the interviews, two interviewers (M.L and B.H.) had a working relationship with some participants, either in a hospital or in a pharmacy. |
| Participant knowledge of  the interviewer | 7 | What did the participants know about the researcher? e.g. personal goals, reasons for doing the research. | Participants were told that the interviews were conducted to collect data on improving antibiotic allergies registrations. |
| Interviewer characteristics | 8 | What characteristics were reported about the interviewer/facilitator? e.g. Bias, assumptions, reasons and interests in the research topic. | Goal of interviewers was to improve antibiotic allergy registration. |
| **Domain 2: Study design** | | | |
| *Theoretical framework* | | | |
| Methodological orientation and Theory | 9 | What methodological orientation was stated to underpin the study? e.g. grounded theory, discourse analysis, ethnography, phenomenology, content analysis. | Content analysis was used. |
| *Participant selection* | | | |
| Sampling | 10 | How were participants selected? e.g. purposive, convenience, consecutive, snowball | Purposive sampling was used. |
| Method of approach | 11 | How were participants approached? e.g. face-to-face, telephone, mail, email | Participants were approached through mail and face-to-face. |
| Sample size | 12 | How many participants were in the study? | 34 |
| Non-participation | 13 | How many people refused to participate or dropped out? Reasons? | 0 |
| *Setting* | | | |
| Setting of data collection | 14 | Where was the data collected? e.g. home, clinic, workplace. | Interviews were conducted with participants at their workplace. |
| Presence of non-participants | 15 | Was anyone else present besides the participants and researchers? | No other persons were present. |
| Description of sample | 16 | What are the important characteristics of the sample? e.g. demographic, data, date | 10 PCP’s, 4 MS, 11 ECP’s, 5 elderly cares nurses and 4 PH’s participated of whom 56% was female and 53% had more than 10 years’ experience. |
| *Data collection* | | | |
| Interview guide | 17 | Were questions, prompts, guides provided by the authors? Was it pilot tested? | The semi-structed interview is included as supplement 3. A pilot interview was performed. |
| Repeat interviews | 18 | Were repeat interviews carried out? If yes, how many? | 34 |
| Audio/visual recording | 19 | Did the research use audio or visual recording to collect the data? | All interviews were digitally recorded. |
| Field notes | 20 | Were ﬁeld notes made during and/or after the interview or focus group? | No notes were taken during interviews. |
| Duration | 21 | What was the duration of the inter views or focus group? | An interview took around 20 to 30 minutes |
| Data saturation | 22 | Was data saturation discussed? | Yes, no new information from answers in 3 consecutive interviews |
| Transcripts returned | 23 | Were transcripts returned to participants for comment and/or correction? | Participants were not asked to comment on or correct the transcribed interview or to provide feedback on the outcome |
| **Domain 3: analysis and ﬁndings** | | | |
| *Data analysis* | | | |
| Number of data coders | 24 | How many data coders coded the data? | Two, Karolina K. Braun and Martijn Sijbom. |
| Description of the coding tree | 25 | Did authors provide a description of the coding tree? | No |
| Derivation of themes | 26 | Were themes identiﬁed in advance or derived from the data? | In advance, through the checklist of Flottorp.^†^ |
| Software | 27 | What software, if applicable, was used to manage the data? | AtlasTi, version 8 |
| Participant checking | 28 | Did participants provide feedback on the ﬁndings? | No |
| *Reporting* | | | |
| Quotations presented | 29 | Were participant quotations presented to illustrate the themes/ﬁndings? Was each quotation identiﬁed? e.g. participant number | In table 4. |
| Data and ﬁndings consistent | 30 | Was there consistency between the data presented and the ﬁndings? | Yes |
| Clarity of major themes | 31 | Were major themes clearly presented in the ﬁndings? | Yes |
| Clarity of minor themes | 32 | Is there a description of diverse cases or discussion of minor themes? | Yes |

*Developed from: Tong A, Sainsbury P, Craig J. Consolidated criteria for reporting qualitative research (COREQ): a 32-item checklist for interviews and focus groups. International Journal for Quality in Health Care. 2007. Volume 19, Number 6: pp. 349 – 357

†Flottorp SA, Oxman AD, Krause J, Musila NR, Wensing M, Godycki-Cwirko M, et al. A checklist for identifying determinants of practice: a systematic review and synthesis of frameworks and taxonomies of factors that prevent or enable improvements in healthcare professional practice. Implementation science : IS. 2013;8:35.
