# Supplementary material for: Cues to improve antibiotic-allergy registration: A mixed-method study
Source: PLoS One. 2022 Apr 7;17(4):e0266473. doi: 10.1371/journal.pone.0266473 (PMC8989191; doi:10.1371/journal.pone.0266473)
Supplement: S3 Table — (DOCX) [file pone.0266473.s003.docx]

# S3 Table. Semi-structured interviews

Name of interviewee:

Position:

Institution / practice (solo / group / other):

Years of experience:

Trainer of medical specialist (MS)/ primary care physician (PCP):

Region:

Patient population (Education level / Social Economic Status / Ethnicity / language barrier):

Electronic Medical Record (EMR) System: (His / Hix / OmniHis / Medicom / MicroHis / HIX other?)

**General**

- Regarding the term antibiotic allergy registration, what do you think about it and what are your thoughts / associations? How do you feel about that?

**Registration of antibiotic allergies in practice**

- How do you inquire about antibiotic allergies?
  - Who inquires and records the allergy?
  - Do you inquire standard or only on indication? For example, when registering a new patient in your practice / institution?
  - Are there agreements about registration within your practice?
    - If yes, explain the method.
    - Do you use it?
    - If so, how does it work in practice?
    - Is the working method clear (defined) to you?
    - Who made the agreements?
    - Are the agreements accessible according to you?
    - Are the agreements practical?
    - What are the agreements based on?
    - Are the agreements comparable with other guidelines?
    - How reliable do you think the current working method is?
  - Is information requested from other institutions, if an allergy is reported?
  - How do you register antibiotic allergies (within your institution)?
    - What method do you use for registration?
    - What is registered (for example, means, type of reaction / evaluation, date, time between administration and occurrence, etc)?
  - How can antibiotic allergy information be found in the system (HIS) you use?
    - Warnings when prescribing? Banner? How can details be found?
    - Is a clear distinction possible between an allergy and a side effect?
  - Are you satisfied with your current registration system / method?
    - What are the advantages and disadvantages of the registration system / method with regard to allergy registration?
  - How can you improve the system?
    - Is the improvement feasible?

**Dealing with suspected antibiotic allergy**

- How do you deal with a suspected allergy?
  - With a patient who does not need antibiotics at that time?
  - With a patient who does need antibiotics?

**Problems with registering antibiotic allergies in practice**

- To what extent are incorrect allergy registrations a problem?
- Are you able to judge whether an allergy registration is correct or incorrect?
  - How do you do that?
  - When are you able/ are you not able?
- Do you run into incorrect or incomplete allergy registrations at your institution / practice?
  - Which one?
  - Incomplete / incorrect?
  - To what extent?
  - What will be done if there is doubt about whether or not an allergy registration is justified?
  - Do you ever leave allergy registration in doubt?
- What do you think are possible causes of incomplete / incorrect registrations?
- What would be the solutions?
- Have previous actions been taken to improve registrations? Which?
- How could you check whether a registration is correct?
  - Is it feasible?

**Delabeling of incorrect allergies**

- What do you do if there is doubt of an allergy registration is incorrect?
- When will an allergy be sorted out?
- Would you be willing to remove an allergy registration from an EMR? What problems do you encounter?

**Communication about allergies with other institutions**

- Is information about allergies shared with other healthcare institutions / providers?
  - And how?
  - Is there a need for that: how / who?
  - Is an allergy in a referral letter included as standard in the episode list?
- How could communication be improved?
- Is there ever feedback from another healthcare provider that the patient has an (alleged) antibiotic allergy?
  - How do you deal with this? (check EMR/ enter registration / ask the patient?)
  - Do you sometimes communicate an (alleged) incorrect antibiotic allergy registration to other heathcare providers?
- How is communication between healthcare providers going?
  - What is going well?
  - What is not going well?
  - What do you think is needed to improve communication about antibiotic allergy?

**Patient perspective**

- To what extent does the patient's presentation of symptoms affect the interpretation and registration of a suspected antibiotic allergy?
- Which factors (knowledge / education / experience of illness) determine the presentation of the symptoms?
- To what extent do you think previous experiences and preferences of patients determine the process of antibiotic allergy registration? (Explain this).
- Are there other patient-related factors that can influence registration?(language barrier, culture, media, guidelines from abroad)
- Could patient explanation about antibiotic allergy contribute to correct registrations?

**Possible areas for improvement regarding the registration of allergies**

- What suggestions do you have to improve registrations? (Training / ICT / Communication / documentation / protocols / website)
- How do you estimate the chance of improvement? (is it feasible? Realistic?)
- What could hinder or facilitate the improvement? (finance, knowledge, time, facilities, opinion / belief / habit and cognitions, management, effort)?
- Are you motivated to improve the allergy registration system in your practice?

**Need for training / guidelines**

- Is there a need for training in the field of allergies / communication / skills? (do you need it yourself?)
  - Focus on which aspects?
  - Which form? (e-learning, classroom, webinar)
- Is there a need for general guidelines with regard to allergy registration?
